# Identifying Contextual Factors and Strategies for Practice Facilitation in Primary Care Quality Improvement Using an Informatics-Driven Model: Framework Development and Mixed Methods Case Study

**DOI:** 10.2196/32174

**Published:** 2022-06-24

**Authors:** Jiancheng Ye, Donna Woods, Jennifer Bannon, Lucy Bilaver, Gayle Kricke, Megan McHugh, Abel Kho, Theresa Walunas

**Affiliations:** 1 Feinberg School of Medicine, Northwestern University Chicago, IL United States

**Keywords:** quality improvement, practice facilitation, primary care, mixed-methods, practice facilitator, informatics, electronic health record, implementation science, implementation, challenge, strategy, framework, perspective

## Abstract

**Background:**

The past decade has seen increasing opportunities and efforts to integrate quality improvement into health care. Practice facilitation is a proven strategy to support redesign and improvement in primary care practices that focuses on building organizational capacity for continuous improvement. Practice leadership, staff, and practice facilitators all play important roles in supporting quality improvement in primary care. However, little is known about their perspectives on the context, enablers, barriers, and strategies that impact quality improvement initiatives.

**Objective:**

This study aimed to develop a framework to enable assessment of contextual factors, challenges, and strategies that impact practice facilitation, clinical measure performance, and the implementation of quality improvement interventions. We also illustrated the application of the framework using a real-world case study.

**Methods:**

We developed the TITO (task, individual, technology, and organization) framework by conducting participatory stakeholder workshops and incorporating their perspectives to identify enablers and barriers to quality improvement and practice facilitation. We conducted a case study using a mixed methods approach to demonstrate the use of the framework and describe practice facilitation and factors that impact quality improvement in a primary care practice that participated in the Healthy Hearts in the Heartland study.

**Results:**

The proposed framework was used to organize and analyze different stakeholders’ perspectives and key factors based on framework domains. The case study showed that practice leaders, staff, and practice facilitators all influenced the success of the quality improvement program. However, these participants faced different challenges and used different strategies. The framework showed that barriers stemmed from patients’ social determinants of health, a lack of staff and time, and unsystematic facilitation resources, while enablers included practice culture, staff buy-in, implementation of effective practice facilitation strategies, practice capacity for change, and shared complementary resources from similar, ongoing programs.

**Conclusions:**

Our framework provided a useful and generalizable structure to guide and support assessment of future practice facilitation projects, quality improvement initiatives, and health care intervention implementation studies. The practice leader, staff, and practice facilitator all saw value in the quality improvement program and practice facilitation. Practice facilitators are key liaisons to help the quality improvement program; they help all stakeholders work toward a shared target and leverage tailored strategies. Taking advantage of resources from competing, yet complementary, programs as additional support may accelerate the effective achievement of quality improvement goals. Practice facilitation–supported quality improvement programs may be opportunities to assist primary care practices in achieving improved quality of care through focused and targeted efforts. The case study demonstrated how our framework can support a better understanding of contextual factors for practice facilitation, which could enable well-prepared and more successful quality improvement programs for primary care practices. Combining implementation science and informatics thinking, our TITO framework may facilitate interdisciplinary research in both fields.

## Introduction

Practice facilitation is an implementation and coaching strategy that aims to develop the capacity of primary care practices to achieve sustained quality improvement (QI) and to address gaps in the implementation of interventions [1]. There is a growing body of evidence suggesting that QI programs that use practice facilitation can produce meaningful and positive change in primary care practices [2,3], including improvements in chronic disease processes and outcome measures for diabetes, asthma [4], cardiovascular disease, and cancer [5]. In addition, practice facilitation interventions that combine audits and feedback, educational materials, and system support are more effective than interventions that use a single approach [6], and they can also lead to a more learning-focused culture, improved work environment, and greater levels of teamwork [7]. Finally, practice facilitators, individuals who are trained to provide QI coaching, can help practices engage in QI activities and develop capacity for continuous QI [1]. Given the complexity and changeability of primary care practices, understanding the context, enablers, barriers, and strategies for implementation of practice facilitation–supported QI programs may help to drive their adoption. Although previous studies have investigated the perspectives of practice leaders and practice facilitators, [8,9] few have incorporated the perspectives of practice staff, who have different roles in the practice. A framework that integrates their feedback, experiences, and strategies with implementation science, technology, and human factor elements is essential to developing effective practice facilitation strategy models [10].

This study aims to design and develop a framework that identifies contextual factors, challenges, and strategies that impact practice facilitation, implementation of QI interventions, and clinical measure performance.

### Framework Development Methods

**Figure 1:**
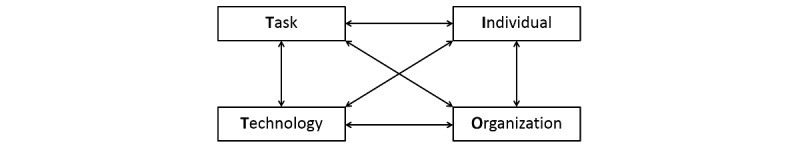
The task, individual, technology, and organization (TITO) framework.

We designed and developed the “task, individual, technology, and organization” (TITO) framework (Figure 1) by combining the “fit between individual, task, and technology” (FITT) model [11] and the “systems engineering initiative for patient safety” (SEIPS) model [12]. The FITT model is often used to understand information technology (IT) adoption, while SEIPS is a theoretical model rooted in human-centered systems that provides a framework for understanding the structures, processes, and outcomes in health care and the relationships between these factors. The SEIPS model has been used to understand or design sociotechnical systems and has supported evaluation, planning and research activities [13]. The components in the SEIPS model include “person,” “organization,” “technologies,” “tasks,” “environment,” “process,” and “outcomes” [12]. The key stakeholders (practice leaders, practice staff, and practice facilitators), informatics researchers, and implementation scientists on the research team collaborated on the participatory workshops to develop a theory-driven framework with testable integration of the elements involved in the study. We discussed the overlaps between the two models and the unique characteristics of quality improvement research. Based on this discussion, we developed TITO by combining the FITT and SEIPS models. All the stakeholders pointed out that health IT (HIT), such as electronic health record (EHR) systems, was important for QI programs. For example, HIT includes data collection for quality measurement, patient outcome monitoring, and intervention implementation [14]. Primary care is an essential part of healthy communities. With QI programs poised to motivate clinicians to improve care quality, investment is needed to ensure that HIT used by clinicians delivers credible data on clinical quality and has the functionality necessary to inform QI efforts in addition to other purposes, such as external reporting for payment, without adding to already high burdens [15]. The research team also conducted literature reviews and multiple conversations with the research team to clarify the terminology and definitions.

The FITT framework enmeshes factors related to the organization of a setting as an intrinsic part of user attributes. However, the organizational context is a critically important factor that affects both practice facilitation and intervention implementation. The “organization” dimension aids the assessment of factors related to the context in which users, tasks, and technology operate. The distinction of “organization” as a separate dimension is necessary, as this could be where key differences between different sites and settings lie. In QI, practice facilitation, or implementation science, organizational factors, such as organizational culture, readiness to engage, and capacity for change, do not fit well into either the individual, task, or technology domains. After recognizing the 4 key domains, we conducted additional literature searches, fine-tuned the domain definitions, summarized what was known about them, proposed ways to measure each domain’s use, and provided examples to increase understanding of what the domains included. Once these documents were drafted and discussed by the research team, a meeting was arranged to present each domain and discuss ways to identify questions and solicit suggestions. TITO is an informatics-driven framework based on systems thinking that can be used in various types of implementation research, such as evaluating, reporting, and synthesizing implementation studies [16].

Table 1 demonstrates the components and constructs of the TITO framework. In TITO, the “task” domain comprises the entirety of tasks and working processes (eg, data extraction and QI reports) that have to be completed by practice leaders, staff, and practice facilitators, and includes care processes, information flow, and process improvement activities. “Individual” represents key stakeholders, including practice leaders, staff, practice facilitators, and patients, as well as their physical and psychological characteristics, education, skills, knowledge, motivation, and needs. “Technology” comprises the interaction of various tools (eg, EHRs, telehealth, online training, computerized provider order entry, and medical devices) needed to accomplish the given tasks and includes electronic and digital tools, tools used by individuals to execute QI tasks, such as paper-based educational materials, and human-factor characteristics (usability, functionality, integration, and availability). Finally, “organization” includes practice culture, leadership, mission, resources, social relationships, supervisory and management style, performance evaluation, rewards and incentives, and the capacity for leading changes. Thus, the TITO framework bridges informatics and implementation science to create a testable framework for future practice facilitation projects, QI initiatives, and health care intervention implementation studies. The framework can be used to organize and analyze complex multilevel factors that impact program success.

**Table 1:** Components and constructs of the task, individual, technology, and organization (TITO) framework.

Domains	Examples of components and constructs
Task	General quality improvement work (data extraction and quality improvement reports), care processes, information flow, and process improvement activities
Individual	Practice leaders, practice staff, practice facilitators, physical and psychological characteristics, education, skills, knowledge, motivation, and needs
Technology	Tools (electronic health records, telehealth, online training, computerized provider order entry, and medical devices), paper-based educational materials, and human-factor characteristics (usability, functionality, integration, and availability)
Organization	Practice culture, leadership, mission, resources, social relationships, supervisory and management style, performance evaluation, rewards and incentives, and capacity for leading changes

This paper presents a case study of an application of this framework and describes context, enablers, and barriers in a primary care practice that participated in a practice facilitation–supported QI study. This case study includes perspectives from 3 key stakeholders to comprehensively examine the TITO framework, shows how each domain in the system interacts and impacts the others, and demonstrates how the framework can be used to summarize contextual factors and strategies for project success.

### Case Study Implementation Methods

#### Healthy Hearts in the Heartland Study

The Healthy Hearts in the Heartland (H3) study aimed to examine the role of practice facilitation in improving 4 cardiovascular clinical quality measures in small primary care practices in Illinois, Indiana, and Wisconsin as part of the Agency for Healthcare Research and Quality-funded EvidenceNOW: Advancing Heart Health in Primary Care program [7]. The H3 study recruited 226 small- and medium-sized primary care practices, which were randomized into 4 study waves that determined when they would start receiving facilitation support. Table S1 in Multimedia Appendix 1 shows the characteristics of the 226 practices. Practice-tailored QI interventions were implemented over a 12-month period, followed by a 6-month sustainability phase. The 4 targeted clinical quality measures included aspirin for ischemic vascular disease, blood pressure control, cholesterol management, and smoking cessation (ABCS) [3]. The QI interventions provided by the H3 study are also shown in Table S2 of Multimedia Appendix 1. Examples include reminders to order aspirin for primary prevention in appropriate patients, orders, patient instructions, patient education for home blood pressure monitoring, reminders to order prescriptions for patients with diabetes, and patient education on smoking cessation [3,17]. Full study details and practice characteristics have been described by Ciolino et al [3].

#### Practice Leaders

Practice leaders were individuals at the practice who were most familiar with the intervention and were generally physicians or QI managers [18]. They were the champions of study implementation and assisted with the whole process of practice facilitation. Practices participating in the H3 study committed personnel time for transformation activities and data transfers for evaluation. Practice leaders monitored and managed the following activities: survey completion, engagement with H3 staff to extract data through EHR reports, troubleshooting or validating data extraction, and manual chart review.

#### Practice Facilitators

Practice facilitators are trained individuals who help practices develop the capacity to make meaningful changes designed to improve patients’ outcomes [19]. Their work includes coaching on practice enhancement methods to facilitate system-level changes. In the H3 study, practice facilitators did the following work: conduct individual biweekly interaction with sites; train clinicians and office staff on QI methods and evidence-based tools to help implement interventions; facilitate modifications to the site’s EHRs to enable systems support for ABCS measurement and monitoring; routinely engage the practice site to implement data reports to facilitate monitoring of quality performance; extract ABCS data and review data with site staff; and document intervention tracking surveys [3].

#### Practice Staff

Practice staff are individuals (eg, clinicians, medical assistants, and front desk staff) who work interactively with practice facilitators to conduct the intervention activities [20]. They received structured training and coaching on clinical topics and QI strategies related to heart health. They also worked with practice facilitators to design and implement QI plans and interventions (shown in Table S2 of Multimedia Appendix 1).

#### Case Selection

To evaluate the TITO framework, we selected a practice from the H3 study that demonstrated an above-average improvement in performance on the ABCS measures from baseline to 12 months and follow-up performance until 18 months. This practice also performed higher than average on the implementation of QI interventions and was considered to have similar characteristics to the average practice in the H3 study across the following dimensions: (1) it had 2 to 5 clinicians, (2) it used the Epic EHR system, and (3) it was not a federally qualified health center, so it could be considered a representative practice.

Table 2 presents the characteristics of the clinical and implementation outcome measurements in this practice. The numerators (n; the number of patients meeting the ABCS criteria) and denominators (N; the total number of eligible patients at the practice for a given criterion) for each of the ABCS measures were generated from native EHR reports.

**Table 2:** Clinical outcome measures and implementation performance of quality improvement interventions.

Measures	Baseline	12 months	18 months
Aspirin use for at-risk individuals, n/N (%)	12/12 (100)	25/26 (96)	13/13 (100)
Blood pressure control, n/N (%)	365/415 (88)	300/339 (89)	289/338 (86)
Cholesterol management, n/N (%)	23/30 (77)	231/287 (80)	12/13 (92)
Smoking cessation, n/N (%)	127/154 (82)	188/196 (96)	1626/1661 (98)
Number of implemented interventions	19	33	34

### Ethics Approval

This study was approved by the Northwestern University Institutional Review Board (STU00201720 and STU00202126). Written consent was obtained from all participants through the H3 study, which was an umbrella study.

### Mixed Methods Approach

This case study applied a mixed methods approach to obtain a greater understanding of the impact of practice facilitation on QI programs, the contextual factors that enabled improved health care quality [21], the experiences of the 3 different types of stakeholder we included, and to help explain the meaning of the data and the forces that facilitated improvement in a qualitative manner [22,23]. Qualitative analyses were conducted by analyzing transcripts from semistructured interviews with practice leaders, staff, and practice facilitators to obtain their perspectives on the implementation of the QI program overall and their approaches to specific interventions. The interviews with the practice leaders and practice facilitators were conducted during the H3 study, and interviews with practice staff were conducted after the study was completed. Quantitative analyses were based on the data from practice facilitation activities, practice surveys, and staff surveys that were collected during the H3 study.

### Qualitative Data Collection and Analysis

We conducted in-depth interviews with the practice leader, the practice facilitator, and 2 practice staff members to understand their experiences and perspectives on the H3 study and to identify and organize contextual factors that impacted QI initiatives. All interviews followed a semistructured protocol (Table S3 of Multimedia Appendix 1). All interview participants had actively interacted with the H3 study.

The interviews, which were conducted by telephone, were audiotaped and transcribed. The interviews with practice staff, which were also audiotaped and transcribed, were conducted on Zoom (version 5.0) [24]. We integrated all the transcribed responses and conducted open coding and axial coding to analyze the data [25]. Two researchers (JY and JB) open-coded the interview data together to identify each instance in which participants talked about their experiences with and attitudes toward the H3 initiative. The 2 researchers then conducted axial coding and grouped open codes that were conceptually similar. Axial coding is a qualitative research technique that involves relating data together in order to reveal codes, categories, and subcategories grounded within the participants’ data [25]. For example, the category “practice culture” includes statements about a practice’s organizational culture and mission; “practice facilitation” include statements describing the workflow and tasks related to practice facilitation; and “patient related barriers” includes “barriers from patients’ social determinants of health and other characteristics.” We resolved discrepancies and developed a consensus codebook encompassing 16 distinct codes (Table 3). The remaining transcripts were then evenly divided between the 2 researchers and coded independently following the codebook [26].

After completing axial coding, the two researchers met and collectively identified preliminary themes. Themes that lacked representation in the data were dropped and similar themes were combined [27]. The final themes were finalized via consensus to represent the most salient perspectives of the participants. Following the proposed TITO framework, we grouped these themes into 4 categories: task, individual, technology, and organization. Under each category, we analyzed the data from 3 stakeholders: practice leader, staff, and practice facilitator.

**Table 3:** Healthy Hearts in the Heartland qualitative analysis codebook.

ID		Code	Definition
**10—Organization**			
	10-1	Communication	Statements about the communication among leaders, staff, and practice facilitators.
	10-2	Resource sharing	Statements about taking advantage of resources from other programs.
	10-3	Practice culture	Statements about a practice’s organizational culture and mission.
	10-4	Capacity for change	Statements about support and mechanisms for making organizational change.
	10-5	Competing priorities	Statements about competing programs or clinical tasks that impact a practice’s engagement.
	10-6	Lack of staff	Statements about a practice lacking personnel for completing the study.
**20—Tasks**			
	20-1	Education and training	The instructions and support that practice facilitators provide for practice.
	20-2	Practice facilitation	Statements describing the workflow and tasks related to practice facilitation.
	20-3	Workload	Burdens on a practice during the quality improvement implementation.
**30—Technology**			
	30-1	Electronic health record capacity	Functionality of the electronic health record system to support the quality improvement study practice facilitation.
	30-2	Resources infrastructure	Statements about electronic or paper resources for practice facilitators and the practice.
	30-3	Quality improvement report	Capacity and challenges for generating quality improvement reports.
**40—Individuals**			
	40-1	Buy-in	Statements about practice leaders, staff, and the practice facilitator’s engagement with the study.
	40-2	Practice facilitator’s strategy	Statements describing the practice facilitator’s skills and approaches that better support practice facilitation.
	40-3	Patients related barriers	Barriers from patients’ social determinants of health and other characteristics.
	40-4	Provider’s mixed opinions.	Statements about providers’ mixed opinions on the guidelines provided by the study team.

### Quantitative Data Collection

#### Practice Facilitation Activities

During the H3 study, practice facilitators documented observations and field notes (eg, coaching activities and degree of implementation success) in standardized fields using the H3 Facilitation Activity and Intervention Tracking System (FACITS) [28]. Data in FACITS included dates of initiation and completion of relevant QI implementation outcomes, the amount of time practice facilitators spent with each practice, and activities performed during practice visits.

#### Practice Survey and Staff Survey

Practice surveys were completed by designated office personnel who had good insight into the clinical operations of the practice [29]. We only included records with complete answers to survey questions by the same personnel at baseline, at 12 months, and at 18 months.

The H3 study incorporated the Change Process Capability Questionnaire (CPCQ) in the practice survey. The CPCQ includes 14 items assessing the extent to which a practice has used specific QI strategies to improve cardiovascular preventive care and evaluates a practice’s resiliency and capacity for change [30]. The scale has been previously validated in small practices, is reliable in measuring practice use of QI strategies, and correlates well with changes in practices and care quality outcomes [31,32]. The CPCQ score was computed as the sum of the items, ranging from −2 (strongly disagree) to 2 (strongly agree). The overall score of the 14 items ranged from −28 to 28 [33]. Higher scores indicated greater use of QI strategies.

## Results

### Practice Characteristics

The selected practice had 5 clinicians (including medical doctors, nurse practitioners, and physician assistants). Before participating in the H3 study, there were no major changes at the clinic (eg, implementation of a new or different EHR system, loss of staff or managers, or moving to a new location). The practice was not in a designated medically underserved area or supporting a medically underserved population as defined by the Health Resources and Service Administration. This was a multi-specialty practice owned by a large health care system and was neither accredited as a patient-centered medical home nor a part of an accountable care organization [34]. The practice’s mission was to address chronic diseases and health disparities; the practice had participated in other QI programs, such as the WISEWOMAN (Well-Integrated Screening and Evaluation for Women Across the Nation) study [35], which shared similar goals as the H3 study, such as management and support of patients with hypertension.

### Staff Working Status

Table 4 illustrates the number of practice members and their combined full-time equivalent (FTE) for each type of staff. FTE is the ratio of the total number of paid hours during a period divided by the number of working hours in that period (eg, one staff member working full-time and another working half time would be 2 staff and 1.5 combined FTE) [36]. FTE is often used to measure a staff member’s involvement in a project or to track cost reductions in an organization [37].

**Table 4:** Staff working status in the practice. Some clinical staff were part-time or volunteers.

Types of staff	Value, n	Combined full-time equivalent
Clinicians, including medical doctors, doctors of osteopathic medicine, nurse practitioners, and physician assistants	4	2.8
Clinical staff providing direct patient care, including registered nurses, licensed practical nurses, medical assistants, and certified medical assistants	5	5
Office staff supporting practice operations but not involved directly with patient care, including receptionists, billing staff, and data analysts	3	3
Social workers or licensed social workers	1	1

### Practice Facilitation Activities

In total, the practice facilitator conducted 39 practice facilitation activities at this practice. The total time of the activities was 805 minutes. The mean time for each activity was 57.5 (SD 26.8) minutes. Among the 39 practice facilitation activities, 11 were on site while 28 were remote. Regarding the encounter type, 20 activities were categorized as “check-in with phone or email,” 16 as “QI meeting,” and 3 as “other” (eg, intervention documentation or extracting data).

### CPCQ Scores

The mean CPCQ score at baseline was 0 (SD 1.18); at 12 months it was 1.14 (SD 0.36), and at 18 months it was 1.86 (SD 0.36). The CPCQ results demonstrated good sustainability of improvement and capacity for leading changes at this practice. In interviews, the staff also reported that the practice had been continuing with many of the suggestions and guidance they received from the H3 study and had continued to show improvement in the ABCS outcomes.

### Participants’ Feedback Summary

We analyzed and mapped the experiences of participants with the H3 study and their attitudes toward it onto the proposed TITO framework. Under each domain, we analyzed the practice survey, staff survey, and interviews. Since the practice leaders, staff, and practice facilitators had different roles in the H3 study, we examined their perspectives separately. Table 5 outlines the participants’ feedback on the H3 study, based on the TITO framework, as an example of how to organize, conceptualize, and examine these contextual factors and strategies.

To demonstrate the 4 domains of the TITO framework, we will illustrate the findings from this case study in more detail to serve as an example for future studies to organize, conceptualize, and examine these contextual factors and strategies. Future studies may have different constructs under each domain.

**Table 5:** Summary of participant feedback on the Healthy Hearts in the Heartland study, based on the TITO (task, individual, technology, organization) framework.

Role	Task	Individual	Technology	Organization
Practice facilitator	Enablers: supported practice with QI^a^ measures and intervention implementation. Barriers: workload and complexity of the QI program tasks.	Enablers: providers were willing to make changes if they found value. Barriers: providers had mixed opinions on some guidelines.	Enablers: high-quality EHR^b^ system; inventory for personalized community resource referral list (Health Rx). Barriers: none identified.	Enablers: well-prepared with rich resources and support from a large health care system. Barriers: small practice; lack of staff; competing priorities.
Practice leader	Enablers: scheduled monthly meeting; met with PF^c^ and passed on information to medical assistants and medical doctors. Barriers: workload.	Enablers: interested in improving and offering better services to patients; worked well with the PF and staff. Barriers: patients’ social determinants of health; patient engagement issues; time pressure	Enablers: used EHR system to generate reports on QI measures. Barriers: hard copies of instructions and information were not appropriate.	Enablers: practice culture facilitated positive change and improvement. Barriers: none identified.
Practice staff—nurses	Enablers: the program was helpful for their routine work. Barriers: some guidelines differed from those used in training at the practice.	Enablers: buy-in to the intervention and coaching activities; the program provided a great deal of useful information that aligned with ongoing work; active engagement and buy-in to the QI program. Barriers: patient compliance.	Enablers: satisfaction with the EHR system; regular reports kept them on track. Barriers: none identified.	Enablers: the program aligned well with the practice’s mission. Barriers: none identified.
Practice staff—program coordinator	Enablers: coordination between providers and QI programs; reaching out to patients; Spanish medical interpreter. Barriers: workload; lack of effective facilitation workflow.	Enablers: the team recognized the value of the program. Barriers: patient health disparities, due to language, immigration status, or transportation issues.	Enablers: support from the affiliated large health care system; satisfaction with the EHR system. Barriers: none identified.	Enablers: the program aligned well with the practice’s mission and ongoing work. Barriers: competing programs.

^a^QI: quality improvement.

^b^EHR: electronic health record.

^c^PF: practice facilitator.

### Tasks

#### Practice Facilitation

Even though the practice leader said that QI practice facilitation “was not a main priority of the practice,” the practice leader added, “it was important that we had this additional help.” The practice leader considered that the H3 study fit well with the practice’s own development plan, provided needed assistance, and gave them a push to better work with resources. The leader engaged in the monthly meetings and, along with the facilitator, sat down and talked about how things were going and what could be improved. The facilitator offered suggestions and the best practice evidence that they found helpful given the current work. The leader thought that “getting an outsider’s perspective on improvement is helpful.”

#### Intervention Implementation

The practice indicated that they wanted to implement all the H3 study interventions at the start of the study. For measures like smoking cessation, since most of the patients in this practice did not smoke, it was easy to achieve high-level performance. Cholesterol management interventions overlapped with another ongoing program in this practice, which allowed the practice to take advantage of resources. To implement the interventions, this practice’s strategy was to take it one step at a time. They first worked on smoking cessation, then blood pressure control. Specifically, they focused on measures that they were struggling with. The leader said that because the practice is small, “It’s easy to get distracted [by clinical work], but H3 has helped the clinic focus on quality improvement.”

#### Individual

##### Patient-Related Factors

Patients in this practice had challenges pertaining to social determinants of health [37]. Most were immigrants and refugees with low incomes. About 80% were primarily Spanish-speaking. The practice leader said that “patient engagement is a problem” and “transportation and work (cannot take off work) also interfere with access to care.” Given these circumstances, the leader said, “If we think we will only see the patient once, we try to take the time to emphasize what they need to do. We also try to do all the lab work during that visit.” In addition, providing hard-copy information about quitting smoking did not work well, since patients seldom read the information. The reasons included the language barrier and low interest.

##### Practice Facilitator

The practice facilitator for this practice had prior social work experience. The practice facilitator developed a good relationship with the practice leader and staff. The practice members trusted the practice facilitator and actively reached out with questions. If they did not see improvement, the practice facilitator remained positive and encouraging. The practice facilitator said, “If we’re not improving, maybe we're not trying the right interventions. We're kind of working on it together.” The practice facilitator “never forced staff members to do something they did not want.” Once the practice made improvement, the practice facilitator would “attribute the improvement to the staff.”

The practice facilitator developed the following practice facilitation working strategies: (1) after each visit, compiling a summary email that included key takeaways and next steps; (2) scheduling the time for the next meeting; (3) documenting and summarizing the meeting and what was planned for the next visit in the FACITS; (4) reviewing the previous meeting’s summary prior to the next meeting and recalling what they would be talking about; and (5) bringing additional materials or information that might be helpful.

The practice facilitator always respected the personnel in the practice, and said, “Let them lead. Don't want them to feel like you're not listening to them by reintroducing them to something they are already aware of” [38]. The practice facilitator formulated instructions and made sure staff knew what to do step by step. The practice facilitator also developed several effective approaches to improving engagement: (1) presenting in person and not letting the practice forget about the study because of competing priorities (the practice facilitator said, “Constant presence in a very positive way. If I ignore H3, no one else is going to pay attention”); (2) writing out definitions of clinical measurements; (3) during meetings, giving providers a paper copy of the definitions and their performance on the QI measures they were tracking so they could take notes.

The quality nurse said the practice facilitator was knowledgeable. If the practice facilitator did not know something, they would reach out to the research team and provide the information to the practice later. Even after the H3 study ended, the staff sometimes still reached out to the practice facilitator with questions regarding some similar tasks that they had worked on before, which reinforced the sustainability of improvement. Regarding resources, the practice facilitator thought the H3 team provided an abundance of resources; however, they found it difficult to find the appropriate material when needed. The practice facilitator’s approach was to use Excel spreadsheets for audits and feedback and present the data in a way that the providers could review in a structured manner. Even so, the practice facilitator still thought that it would have been helpful to “have more of a tailored menu of ways to present the resources.”

#### Technology

##### EHR system

The EHR system used by the practice during the H3 study was Epic (version 2014, Epic Systems Corporation). The robust features of this system facilitated QI activities. The EHR vendor also helped extract data and clinical quality measures. Data from the practice physically resided in the health system’s data warehouse. The EHR system was certified to meet meaningful use as defined by Health and Human Services/Office of the National Coordinator for Health Information Technology (ONC). The practice was able to incorporate clinical laboratory test results into the EHR as structured data (ie, data were recorded in discrete fields and not in text fields). The practice also had the ability to electronically share patient health information (eg, lab results, imaging reports, problem lists, and medication lists) with other providers, including hospitals, ambulatory providers, and outside labs.

##### QI Measure Report

The practice could generate reports on all four ABCS QI measures at the practice level. There was an IT service provided within the health care system that was responsible for configuring and writing quality reports from the EHRs. It also worked with the practice network, health information exchange, and hospital network to report clinical quality measures.

#### Organization

##### Infrastructure Resources

Although the practice was small, it had many resources; for example, the practice staff noted that through the WISEWOMAN program [39] “a lot of blood pressure work redesigned exam rooms through that project.” The practice was owned by a large health care system to which the practice could refer patients. It also had a very extensive patient assistance program; this program had a full-time staff member dedicated to helping patients apply for medication assistance from pharmaceutical companies.

##### Practice Culture

The practice was open to change and interested in improving and offering better health care services to patients. The CPCQ score in this practice increased after 12 months of practice facilitation and continued to improve during the 6-month sustainment period, which demonstrated the capacity for change and ability to maintain improvement of this practice. The leader and staff welcomed suggestions from the practice facilitator. This culture brought benefits, such as including outside perspectives into their regular meetings and adopting best practices from other practices, as well as providing a consistent external reminder of the importance of the work. All the staff were flexible and open to new ideas and unified in the mission to address health disparities. They were always willing to support patients who faced barriers and were marginalized by the health care system. The practice leader provided strong support, and practice staff were actively engaged in the practice facilitation activities in the H3 study.

##### Staffing Resources

The practice leader and staff felt they had a “lack of staff.” Because it was a small clinic, they had many competing priorities.

##### Successful Experiences, Challenges, and Recommended Solutions

We also used the TITO framework to organize successes and challenges within the H3 study and to develop solutions to address these challenges. The results are presented in Table 6.

**Table 6:** Summary of successful experiences, challenges, and recommended solutions.

Aspects	Successful experiences	Challenges	Recommended solutions
Task	Monthly meetings and discussing new strategies; everyone had a voice. Took advantage of resources from other ongoing/finished programs. Small group sessions brought back to a larger group. History of patient outreach. Informative training and education materials. Structured instructions. Interventions fit the practice’s development direction. Provided materials in the language that most patients spoke (Spanish).	Providers had mixed views on some guidelines. High workload.	Brainstorming sessions and discussion. Meeting over the lunch hour and catching up.
Individual	Practice leaders and staff were flexible and open to new strategies. Active engagement. Good relationship among practice facilitator, practice leader, and staff. Effective communication/bidirectional conversation. Practice facilitator was positive and encouraging. Quality nurse was focused.	Patients’ social determinants of health and health disparities.	Providing culturally competent and linguistically appropriate information about health. Incentivizing and supporting practice facilitation through improved payment models (eg, incentivize providers based on the time they work on the project and whether their progress is reasonable).
Technology	Well-organized electronic health record infrastructure. Inventory for personalized community resource referral list (Health Rx) enabled the practice facilitator to check what was needed. Owned by a large health system; health information technology resources were shared.	Too many resources (eg, human and paper tools) for the practice facilitator.	Making available resources well-organized and easy to navigate.
Organization	Complemented other programs. Leadership support. Focused on the mission. Understood the importance of quality improvement. High level of collaboration and teamwork.	Competing programs. Limited time. Lack of staff.	Complementation with resources from different programs.

## Discussion

### Study Overview

This study designed, developed, and piloted the TITO framework, which combined the FITT and SEIPS frameworks to understand the impact of practice facilitation on clinical measure performance and the implementation of QI interventions. We present the application of this informatics-driven framework as the analysis of a case study, describing the context, enablers, barriers, and strategies of a primary care practice that participated in a practice facilitation–supported QI program. We analyzed and compared different perspectives from 3 key stakeholders using systems thinking, which allowed for comprehensive examinations of where their perspectives aligned or diverged.

### Informatics-Driven Implementation Framework

The TITO framework provides a more comprehensive description of the 4 components of QI initiatives using systems thinking (task, individual, technology, and organization). This framework could enable further development of specific measures within these domains to create a standardized template to build tailored implementation research logic models [40] and better comparisons across QI programs [41]. Because TITO was developed based on informatics perspectives and systems thinking, it may foster a common language and complement other theoretical models [42], including the Consolidated Framework for Implementation Research (CFIR) framework [43]. The tradeoff may not be significant given that qualitative results are not often considered generalizable, but rather “transferrable.” The CFIR is qualitatively different from implementation models derived from the informatics field. Combining informatics-based thinking and implementation science models may combine the advantages of both approaches and introduce benefits for a wide variety of improvement initiatives, practice settings, and care changes. The TITO framework may provide practical and actionable guidance for different stakeholders in QI programs in primary care. For example, technologies such as EHR systems will bring benefits for tasks like QI measurement reporting. Respectful negotiations and transparent communication between practice facilitators and practice staff can foster “win-win” results. Although we applied the framework in a small primary practice and focused on QI interventions for cardiovascular care, this framework may be helpful for a wide variety of QI initiatives, practice settings, and health care systems [44,45]

### Primary Care Quality Improvement

For this case study, which was an extension of the H3 study, the selected practice provided lessons that may be generalizable to a broader range of primary care practices. From the practice leader’s perspective, notable barriers included patients’ social determinants of health and a lack of staff and time, but there were also outstanding enablers, such as staff buy-in, effective practice facilitation strategies, and shared complementary resources from similar ongoing programs. The practice staff thought the competing programs created a burden on their engagement and routine care. However, taking advantage of the resources from similar ongoing programs could have provided additional support, which may have helped accelerate improvement in the QI initiative. From the practice facilitator’s perspective, the key enablers were the practice’s capacity to make change and the practice culture, while notable barriers included unsystematic facilitation resources. Finally, practice leaders and staff reported benefiting from targeted assistance, such as EHR documentation guidance and connections to reporting tools, resources, and training activities. Practice facilitators, however, reported that limited engagement, busy schedules, and patient characteristics led to challenges.

### Application of the Framework

Leveraging the TITO framework, we identified contextual factors and strategies for practice facilitation in primary care quality improvement in 4 domains: task, individual, technology, and organization. Overall, a successful QI program should fit well within a practice’s existing strategies and mission to enable organization-level improvement and provide appropriate assistance and resources for changes in task-level improvement [46]. In the H3 initiative, most interventions were offered based on the interests of practices in the study and what they were likely to be capable of implementing. Practice facilitation works best when the practice leader and staff actively engage with the practice facilitator, recognize the importance of the study, and agree with the implementation strategies. Effective collaboration and communication among the 3 stakeholders are essential for the successful implementation of practice facilitation and QI intervention.

For small primary care practices, the lack of staff is a major problem [47]. Our findings from this case study suggest that one way to navigate this issue is to focus efforts, implement interventions one at a time, and use resources from other ongoing programs to complement the activity. In addition, HIT can introduce benefits with the right support [48]. An EHR system with effective reporting functionality in combination with technical support from the vendor resulted in clinical-quality-measure reports that were valuable for assessing the success of QI interventions. With a solid technology foundation, sustainable quality improvement efforts, as well as the regular collection and review of clinical measures, were readily achieved. The features illuminated by this case study may be helpful to other small primary care practices seeking to improve clinical performance.

TITO also emphasizes the individual domain; successful interventions in patient populations with health disparities may require adaptation [49]. In some instances, the primary care providers could not provide patients with appropriate care due to challenging engagement issues (eg, transportation, time, and language). This led the practice in this case study to take several actions: (1) emphasize health equity and make every effort to address any patient concerns during their clinical visits; (2) recruit volunteers who could speak the same language as the patients to reach out to individuals that had higher risks; and (3) ensure that health care providers made full use of their time during each patient visit, such as by doing all the necessary clinical care that was applicable during the visit. Although patients typically are not involved in practice facilitation, it would be worthwhile to consider working with patient representatives or using a community engagement process to gather feedback on ways the QI program might best address their needs [46].

Practice facilitators are key liaisons during QI practice facilitation. They must earn trust and buy-in from the practice leader and staff from the beginning of a QI program. Developing effective communication styles and skills will help practice facilitators establish and reinforce a collaborative relationship within which they can implement and foster sustainability of the QI intervention. A commitment to collaboration with humility will go a long way in supporting practices and achieving success [46]. Practice facilitators can use motivational approaches to conduct coaching activities with clinical champions, help the practice initiate QI, facilitate the application of knowledge and QI tools to improve clinical practice, provide informational resources, and motivate practice members to engage in teamwork. Clinical staff may exhibit varying levels of acceptance of program guidelines; the practice facilitator should respect their opinions and invest in time for relationship building to understand their perspectives. In addition, the practice facilitator should use tailored strategies to manage diverse resources and ensure that materials are organized, structured, and accessible for use when needed [50] to increase the efficiency of their approach.

The TITO framework introduces “organization” as an important factor, because this could be where key differences between different sites and settings lie, especially for primary care. The presence of a practice culture with a positive attitude toward change and the absence of a disruptive level of organizational stress can be effective contributors to success. The practice should be open to change and interested in improving and offering better services to patients, regardless of whether there are financial incentives in place. Engagement in QI initiatives is more likely to be productive when practice members actively decide to participate because the QI efforts align with their fundamental values and norms—that is, viewing targeted QI efforts as a way to provide better care to their patients—not just another revenue stream for the practice or a bothersome bureaucratic burden [50]. All the staff should be flexible, open to new ideas, and unified in their commitment to a mission to address health disparities, and practice leaders should provide strong support. With such a culture, sustainable improvement can be maintained regardless of workforce turnover.

### Limitations

There are some limitations to this study. First, the interviews with the practice leader and practice facilitator were conducted during the H3 study, while interviews with staff were conducted after the initiative was completed, which may have introduced recall bias. Even so, we followed up with the practice facilitator, discussed our findings, and resolved discrepancies. Because of the timing of this investigation, we were also able to examine the sustainability of the QI initiative in this practice. Second, since this case study was focused on a single primary care practice, the study observations, results, and conclusions may not be generalizable to a wider group of practices, and the codes and categories generated from our grounded theory approach may be limited in scope. Nevertheless, this practice was selected because it had the same characteristics as most of the other practices in the H3 study, and it could have thus provided valuable lessons and implications for practices within or outside the H3 study. Third, because of the nature of case studies, it was impossible to determine causal relationships; however, our findings could suggest hypotheses for future studies as to what contextual factors are related to success.

### Conclusion

In this study, we designed and developed the TITO framework to identify contextual factors and strategies that impact practice facilitation, clinical measure performance, and implementation of QI interventions. The practice leader, staff, and practice facilitator all saw value in the QI initiative; however, they faced different challenges and used different strategies during the practice facilitation. These challenges and strategies could be clearly defined using the TITO framework. The TITO framework also supports a better understanding of the contextual factors and strategies for practice facilitation and therefore may enable better-prepared and more-successful QI programs in primary care. With the uptake of implementation science and informatics thinking, the TITO framework may facilitate interdisciplinary research in these two fields. The TITO framework will also be a useful and generalizable guideline for future practice facilitation projects, QI initiatives, and health care intervention implementation studies to organize and analyze the complex, multilevel factors that impact the success of the program.

## Appendix

Multimedia Appendix 1 Supplemental.

## Abbreviations

ABCS: aspirin for ischemic vascular disease, blood pressure control, cholesterol management, and smoking cessation
CFIR: Consolidated Framework for Implementation Research
CPCQ: Change Process Capability Questionnaire
EHR: electronic health record
FACITS: Facilitation Activity and Intervention Tracking System
FITT: fit between individual, task and technology
FTE: full-time equivalent
H3: Healthy Hearts in the Heartland
HIT: health information technology
IT: information technology
QI: quality improvement
SEIPS: systems engineering initiative for patient safety
TITO: task, individual, technology, and organization
WISEWOMAN: Well-Integrated Screening and Evaluation for Women Across the Nation
