# Metadata Correction: Identifying Contextual Factors and Strategies for Practice Facilitation in Primary Care Quality Improvement Using an Informatics-Driven Model: Framework Development and Mixed Methods Case Study

**DOI:** 10.2196/40674

**Published:** 2022-07-08

**Authors:** Jiancheng Ye, Donna Woods, Jennifer Bannon, Lucy Bilaver, Gayle Kricke, Megan McHugh, Abel Kho, Theresa Walunas

**Affiliations:** 1 Feinberg School of Medicine, Northwestern University Chicago, IL United States

In “Identifying Contextual Factors and Strategies for Practice Facilitation in Primary Care Quality Improvement Using an Informatics-Driven Model: Framework Development and Mixed Methods Case Study” (JMIR Hum Factors 2022;9(2):e32174) the authors noted one error.

In the originally published manuscript, a footnote was erroneously displayed in the authorship list noting equal contribution only for author Jiancheng Ye.

This footnote has been removed from the corrected manuscript, as no equal contribution should be noted in the authorship list.

The correction will appear in the online version of the paper on the JMIR Publications website on July 8, 2022, together with the publication of this correction notice. Because this was made after submission to PubMed, PubMed Central, and other full-text repositories, the corrected article has also been resubmitted to those repositories.

