# Correction: Identifying Contextual Factors and Strategies for Practice Facilitation in Primary Care Quality Improvement Using an Informatics-Driven Model: Framework Development and Mixed Methods Case Study

**DOI:** 10.2196/84390

**Published:** 2025-10-08

Following the publication of “Identifying Contextual Factors and Strategies for Practice Facilitation in Primary Care Quality Improvement Using an Informatics-Driven Model: Framework Development and Mixed Methods Case Study” [1], concerns were raised regarding the corresponding author Jiancheng Ye’s rate of self-citation within the article’s reference list. Further investigation into this matter identified that numerous self-citations were added to the manuscript following acceptance of the article without adequate disclosure.

All authors were contacted for a response regarding this matter. Author Theresa Walunas indicated that they were unaware of the addition of references post acceptance. Authors Donna Woods, Jennifer Bannon, Lucy Bilaver, Gayle Kricke, Megan McHugh, and Abel Kho did not respond. Corresponding author Jiancheng Ye responded with a scientific justification for each added self-citation within the published article.

Following review of these responses, the JMIR Publications Editorial Office determined that several of the added references were not relevant to the article or were used to support a generic statement where another reference could have been used instead of self-citation. Therefore, the JMIR Publications Editorial Office is issuing this corrigendum to remove the following references:

- Reference 17: Ye J. The role of health technology and informatics in a global public health emergency: practices and implications from the COVID-19 pandemic. JMIR Med Inform 2020 Jul 14;8(7):e19866 [doi: 10.2196/19866] [Medline: 32568725]
- Reference 19: Ye J, Li N, Lu Y, Cheng J, Xu Y. A portable urine analyzer based on colorimetric detection. Anal Methods 2017;9(16):2464-2471 [doi: 10.1039/c7ay00780a]
- Reference 38: Ye J, Orji I, Baldridge AS, Ojo T, Shedul G, Ugwuneji E, et al. Abstract MP19: characteristics and patterns of retention in hypertension care in primary care settings: findings from the Hypertension Treatment in Nigeria Program. Circulation 2022;145 [doi: 10.1161/circ.145.suppl_1.mp19]
- Reference 40: Ye J. The impact of electronic health record-integrated patient-generated health data on clinician burnout. J Am Med Inform Assoc 2021 Apr 23;28(5):1051-1056 [doi: 10.1093/jamia/ocab017] [Medline: 33822095]
- Reference 42: Ye J, Ren Z. Using multivariate models to examine the impact of COVID-19 pandemic and gender differences on health and health care. medRxiv. Posted on September 5, 2021 [doi: 10.1101/2021.09.02.21263055]
- Reference 44: Ye J. Health information system’s responses to COVID-19 pandemic in China: a national cross-sectional study. Appl Clin Inform 2021 Mar 19;12(2):399-406 [doi: 10.1055/s-0041-1728770] [Medline: 34010976]
- Reference 45: Ye J, Yao L, Shen J, Janarthanam R, Luo Y. Predicting mortality in critically ill patients with diabetes using machine learning and clinical notes. BMC Med Inform Decis Mak 2020 Dec 30;20(Suppl 11):295 [doi: 10.1186/s12911-020-01318-4] [Medline: 33380338]
- Reference 46: Ye J, Wang Z, Hai J. Social networking service, patient-generated health data, and population health informatics: national cross-sectional study of patterns and implications of leveraging digital technologies to support mental health and well-being. J Med Internet Res 2022 Apr 29;24(4):e30898 [doi: 10.2196/30898] [Medline: 35486428]
- Reference 54: Ye J, Ma Q. The effects and patterns among mobile health, social determinants, and physical activity: a nationally representative cross-sectional study. AMIA Jt Summits Transl Sci Proc 2021;2021:653-662 [Medline: 34457181]
- Reference 59: Ye J. Pediatric mental and behavioral health in the period of quarantine and social distancing with COVID-19. JMIR Pediatr Parent 2020 Jul 28;3(2):e19867 [doi: 10.2196/19867] [Medline: 32634105]

The JMIR Publications Editorial Office regrets that these issues were not identified prior to publication.

The correction will appear in the online version of the paper on the JMIR Publications website together with the publication of this correction notice. Because this was made after submission to PubMed, PubMed Central, and other full-text repositories, the corrected article has also been resubmitted to those repositories.
