# Supplementary material for: Identifying Contextual Factors and Strategies for Practice Facilitation in Primary Care Quality Improvement Using an Informatics-Driven Model: Framework Development and Mixed Methods Case Study
Source: JMIR Hum Factors. 2022 Jun 24;9(2):e32174. doi: 10.2196/32174 (PMC9269526; doi:10.2196/32174)
Supplement: Multimedia Appendix 1 [file humanfactors_v9i2e32174_app1.docx]

**Identifying** **contextual factors and strategies for practice facilitation in primary care quality improvement using an informatics-driven model: framework development and mixed-methods case study**

Jiancheng Ye^1,3*^, Donna Woods^1,2^, Jennifer Bannon^3^, Lucy Bilaver^1,2^, Gayle Kricke^1^, Megan McHugh^1^, Abel Kho^3,4^, Theresa Walunas^3,4^

1 Center for Education in Health Sciences, Institute for Public Health and Medicine, Feinberg School of Medicine, Northwestern University, Chicago, Illinois, USA

2 Department of Pediatrics, Feinberg School of Medicine, Northwestern University, Chicago, Illinois, USA

3 Center for Health Information Partnerships, Institute for Public Health and Medicine, Feinberg School of Medicine, Northwestern University, Chicago, Illinois, USA

4 Department of Medicine, Division of General Internal Medicine and Geriatrics, Feinberg School of Medicine, Northwestern University, Chicago, Illinois, USA

*Corresponding author; 633 N. Saint Clair St, Chicago, IL 60611, USA;

Email: jiancheng.ye@u.northwestern.edu; Tel: 312-503-3690

**SUPPLEMENTAL TABLES**

**Supplemental Table 1.** Characteristics of 226 practices in Illinois, Wisconsin, and Indiana participating in the H3 initiative

| **Characteristics** | **N (%)** |
| --- | --- |
| **Number of practices by wave** |  |
| Wave 1 | 42 (18.6) |
| Wave 2 | 40 (17.7) |
| Wave 3 | 67 (29.6) |
| Wave 4 | 77 (34.1) |
| **Number of clinicians** |  |
| Solo practice | 67 (29.6) |
| 2-5 Clinicians | 103 (45.6) |
| 6-10 Clinicians | 34 (15.0) |
| 11-15 Clinicians | 10 (4.40) |
| 16 -20 Clinicians | 12 (5.30) |
| **State** |  |
| Illinois | 152 (67.3) |
| Wisconsin | 22 (9.7) |
| Indiana | 52 (23.0) |
| **FQHC** |  |
| Yes | 64 (28.3) |
| No | 162 (71.7) |

FQHC: Federally Qualified Health Center

**Supplemental Table 2.** H3 intervention list (N=35)

| **Intervention ID** | **Category** | **Measure** | **Component** |
| --- | --- | --- | --- |
| 1 | A. Point-of-Care Clinical Decision Support | Aspirin | Reminder to order aspirin/antiplatelet drug for pts with IVD (or CVD) |
| 2 | A. Point-of-Care Clinical Decision Support | BP | Alert staff to a patient with uncontrolled blood pressure |
| 3 | A. Point-of-Care Clinical Decision Support | Cholesterol | Alert for a lipid panel (or cholesterol) in ASCVD (or IVD, or CVD) |
| 4 | A. Point-of-Care Clinical Decision Support | Cholesterol | Alert for a lipid panel (or cholesterol) in diabetes mellitus |
| 5 | A. Point-of-Care Clinical Decision Support | Cholesterol | Alert for a lipid panel in general population (low risk patients) |
| 6 | A. Point-of-Care Clinical Decision Support | Cholesterol | Reminder to order a statin in ASCVD (or IVD, or CVD) |
| 7 | A. Point-of-Care Clinical Decision Support | Cholesterol | Reminder to order statin in diabetic patients |
| 8 | A. Point-of-Care Clinical Decision Support | Cholesterol | Alert to order a statin in pts with LDL 190 |
| 9 | A. Point-of-Care Clinical Decision Support | Cholesterol | Alert to order statin in general population with increased risk (based on a risk calculator) |
| 10 | A. Point-of-Care Clinical Decision Support | Aspirin | Reminder to order aspirin for primary prevention in appropriate patients |
| 11 | A. Point-of-Care Clinical Decision Support | Smoking | Reminder for intervention in tobacco users or smokers |
| 12 | B. Other Clinical Decision Support Activities | BP | Orders/patient instructions/patient education for home BP monitoring |
| 13 | B. Other Clinical Decision Support Activities | Cholesterol | Patient education on cholesterol and/or cholesterol treatment |
| 14 | B. Other Clinical Decision Support Activities | Cholesterol | Standing orders for lipid profiles |
| 15 | B. Other Clinical Decision Support Activities | Smoking | Patient education on tobacco cessation |
| 16 | C. Practice Workflows | BP | Blood pressure measurement protocol |
| 17 | C. Practice Workflows | BP | Blood pressure treatment protocol |
| 18 | C. Practice Workflows | BP | Workflow for patient to report home blood pressures |
| 19 | C. Practice Workflows | Smoking | Tobacco use/smoking assessment part of intake or rooming process |
| 20 | C. Practice Workflows | Smoking | Clinic based tobacco use/smoking interventions |
| 21 | D. Reports on ABCS Performance | Aspirin | Metric for use of aspirin or another antithrombotic therapy in IVD |
| 22 | D. Reports on ABCS Performance | BP | Metric for blood pressure control among patients with hypertension |
| 23 | D. Reports on ABCS Performance | Cholesterol | Metric for cholesterol treatment or control |
| 24 | D. Reports on ABCS Performance | Smoking | Metric for tobacco use assessment and brief intervention |
| 25 | E. Lists of Patients Not Meeting ABCS Measures | Aspirin | List of patients with IVD not meeting aspirin/antithrombotic measure |
| 26 | E. Lists of Patients Not Meeting ABCS Measures | BP | List of patients with uncontrolled blood pressure |
| 27 | E. Lists of Patients Not Meeting ABCS Measures | Cholesterol | List of patients needing cholesterol measurement and/or treatment |
| 28 | E. Lists of Patients Not Meeting ABCS Measures | Smoking | List of tobacco users/smokers |
| 29 | F. Population Management Outreach | Aspirin | Outreach to patients with IVD not on aspirin or another antithrombotic |
| 30 | F. Population Management Outreach | BP | Outreach to patients with uncontrolled hypertension |
| 31 | F. Population Management Outreach | Cholesterol | Outreach to patients who need cholesterol measurement or statin prescription |
| 32 | F. Population Management Outreach | Smoking | Outreach to tobacco users or smokers |
| 33 | F. Population Management Outreach | Cholesterol | Outreach to patients with increased CVD risk who are not on a statin for primary prevention |
| 34 | G.Population Management Community Resources | BP | Referral to community pharmacist for hypertension medication management |
| 35 | G.Population Management Community Resources | Other | Referral to HealtheRx resource |

**Supplemental Table 3.** Interview protocol

| 1.     Date of interview |
| --- |
| 2.     Respondent role and profession |
| 3.     H3 strengths |
| 4.     H3 weaknesses |
| 5.     Easiest interventions |
| 6.     Hardest interventions |
| 8.     External factors that helped |
| 9.     External factors that hindered |
| 10.   Sustainability of changes |
| 11.   Burden |
| 12.   Internal enablers |
| 13.   Internal barriers |
| 15.   Successful experience |
| 16.   Advice for H3 team and other practices |
| 17.   Final thoughts |
